# Canine tumor cross-species genomics uncovers targets linked to osteosarcoma progression

**DOI:** 10.1186/1471-2164-10-625

**Published:** 2009-12-23

**Authors:** Melissa Paoloni, Sean Davis, Susan Lana, Stephen Withrow, Luca Sangiorgi, Piero Picci, Stephen Hewitt, Timothy Triche, Paul Meltzer, Chand Khanna

**Affiliations:** 1 Comparative Oncology Program, Center for Cancer Research, National Cancer Institute, National Institutes of Health, Bethesda, MD, 20892, USA; 2 Genetics Branch, Center for Cancer Research, National Cancer Institute, National Institutes of Health, Bethesda, MD, 20892, USA; 3 Animal Cancer Center, Veterinary Teaching Hospital Colorado State University, Fort Collins, CO, 80523, USA; 4 The Rizzoli Orthopedic Institute, Laboratory of Oncologic Research Bologna, Bologna 40136, Italy; 5 Tissue Array Research Program, Center for Cancer Research, National Cancer Institute, National Institutes of Health, Bethesda, MD, 20892, USA; 6 The Saban Research Institute, Childrens Hospital Los Angeles, University of Southern California, Los Angeles, CA, 90027, USA

## Abstract

**Background:**

Pulmonary metastasis continues to be the most common cause of death in osteosarcoma. Indeed, the 5-year survival for newly diagnosed osteosarcoma patients has not significantly changed in over 20 years. Further understanding of the mechanisms of metastasis and resistance for this aggressive pediatric cancer is necessary. Pet dogs naturally develop osteosarcoma providing a novel opportunity to model metastasis development and progression. Given the accelerated biology of canine osteosarcoma, we hypothesized that a direct comparison of canine and pediatric osteosarcoma expression profiles may help identify novel metastasis-associated tumor targets that have been missed through the study of the human cancer alone.

**Results:**

Using parallel oligonucleotide array platforms, shared orthologues between species were identified and normalized. The osteosarcoma expression signatures could not distinguish the canine and human diseases by hierarchical clustering. Cross-species target mining identified two genes, interleukin-8 (*IL-8*) and solute carrier family 1 (glial high affinity glutamate transporter), member 3 (*SLC1A3*), which were uniformly expressed in dog but not in all pediatric osteosarcoma patient samples. Expression of these genes in an independent population of pediatric osteosarcoma patients was associated with poor outcome (p = 0.020 and p = 0.026, respectively). Validation of *IL-8 *and *SLC1A3 *protein expression in pediatric osteosarcoma tissues further supported the potential value of these novel targets. Ongoing evaluation will validate the biological significance of these targets and their associated pathways.

**Conclusions:**

Collectively, these data support the strong similarities between human and canine osteosarcoma and underline the opportunities provided by a comparative oncology approach as a means to improve our understanding of cancer biology and therapies.

## Background

Osteosarcoma is a highly metastatic cancer of bone seen primarily in pediatric patients. Approximately 800-1,000 children develop osteosarcoma yearly, with peak incidence during the adolescent growth spurt [1]. For patients with localized disease, the use of chemotherapy following surgery, introduced in the 1970s, improved long-term survival from approximately 20% to over 60%. These outcomes have been largely unchanged despite intensification of adjuvant therapy over the last 30 years [2]. For patients who present with metastatic disease, the prognosis is even more grave with survival rates of less than 20% [3]. Advances are needed in our understanding of metastasis biology and therapy to improve outcomes for patients.

An increasingly considered modeling approach in cancer biology and therapeutic development is the study of naturally occurring cancers in pet dogs (referred to as comparative oncology). The features of cancers in pet dogs that may uniquely contribute to our understanding of cancer pathogenesis, progression and therapy have been recently reviewed [4]. Companion (pet) dogs develop osteosarcoma at similar sites as human patients, with identical histology, response to traditional treatment regimens such as surgery and chemotherapy, and proclivity for metastasis [6,7]. Similarly, many of the candidate genes implicated in the pathogenesis or progression of osteosarcoma in children have also been characterized in the canine disease, notably *PTEN*: phosphatase and tensin homolog, *Rb*: retinoblastoma, *ezrin*; villin-2, *c-met*: mesenchymal-epithelial transition factor, *erbB-2*: v-erb-b2 erythroblastic leukemia viral oncogene homolog 2, neuro/glioblastoma derived oncogene homolog (avian) and *p53*: tumor protein 53 [7-12]. The incidence of osteosarcoma in dogs is higher than children, with >10,000 dogs diagnosed yearly [6]. The canine disease is considered to be more aggressive than the human disease. The use of surgery alone is associated with long-term survival in 5% of dogs [5,6,13]. In the era before adjuvant chemotherapy for pediatric patients long-term survival ranged from 17-30% [14,15]. Differences in disease prevalence and the more aggressive disease biology in the dog further argues the opportunity for this approach to inform our understanding of this highly aggressive pediatric cancer. Although limited in scope, pet dogs with osteosarcoma have been effectively integrated into the development of novel treatment approaches for human patients, most notably pioneering limb-sparing techniques [16,17]. In 2005 the first public draft of the canine genome sequence was released [18,19]. This milestone provided the opportunity for dogs with cancer to lend additional insight into the biology of human cancers, and to more rigorously evaluate and translate novel therapies to human trials.

Based on these opportunities we employed a cross-species gene expression approach based on the hypothesis that the identification of conserved and distinctive genes, gene families and functions across cross-species would provide a unique perspective to view the determinants of osteosarcoma biology. Using identical oligonucleotide microarray platforms we compared expression signatures for osteosarcoma primary tumors and normal tissues from both dogs and humans. The similarities between canine and human osteosarcoma were strong. Cluster analysis of 265 orthologous transcripts could not distinguish the cancers based on species alone. These data strengthened the scientific rationale for the inclusion of dogs with osteosarcoma in the study of cancer biology and therapy. Based on the similarities between the expression profiles of human and canine osteosarcoma, we then asked whether the more aggressive biology of osteosarcoma in the canine disease could help identify subsets or candidate genes important in the metastatic progression of osteosarcoma that would have been overlooked if the human disease was studied alone. Four genes were identified that were consistently overexpressed in canine osteosarcoma ("dog-like" genes) but had variable and/or low expression in humans. Evaluation of the dog-like genes in a distinct population of human osteosarcoma patients, which were linked to clinical outcome, confirmed that two genes (*IL-8*, interleukin-8, and *SLC1A3*, solute carrier family 1 (glial high affinity glutamate transporter)) were potentially associated with a more aggressive clinical course in human patients. This is the first non-candidate comparative genomic analysis of spontaneous disease between dog and man. These data support the inclusion of canine osteosarcoma as a clinical intermediary in the study of novel anti-cancer therapeutics destined for use in man. Furthermore, the strength of the similarity between species, coupled with the more aggressive biology of the disease in dogs allowed the identification of new genes that may be relevant targets or predictors of metastatic outcome in human osteosarcoma.

## Results

### Within Species Osteosarcoma Gene Expression Profiling

**Figure 1:**
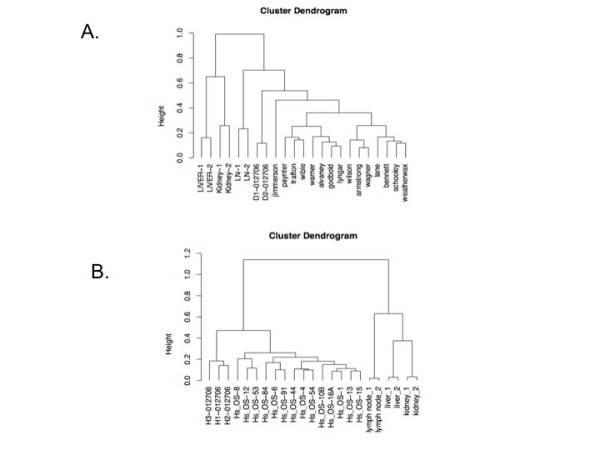
**Single species cluster dendrograms define canine and human osteosarcoma as distinct from normal organs and osteosarcoma cell lines**. Cancer defining gene signatures were generated by calculating the differential expression between canine and human osteosarcoma samples and their respective normal organs using limma (Linear Models for Microarray Data, adjusted p value < 0.01). **A. **The canine cancer signature consists of 3471 genes and **B. **human cancer signature 2705. High level, unsupervised hierarchical clustering conducted in each species separately resulted in osteosarcoma samples clustering together and distinctly from normal tissues and their respective cancer cell lines.

Comparison of osteosarcoma versus normal tissues gene expression in each species was determined using limma software (Linear Models for Microarray Data, adjusted p value < 0.01) [20]. The cancer signatures consisted of 3471 and 2705 probesets in the canine and human analyses, respectively. High level, unsupervised hierarchical clustering conducted in each species separately resulted in osteosarcoma samples clustering together and distinctly from normal tissues. (Figure 1A and 1B) Not surprisingly the normal tissues clustered with organ replicates. Clustering of cancers was not associated with histologic subtype or primary tumor location. Quantitative RT-PCR was used to validate the results of the microarray. The pattern and magnitude of expression for each validation gene (*LGALS3*, galectin 3; *MFAP5*, microfibrillar associated protein 5; *PRKDC1*: protein kinase, DNA-activated, catalytic polypeptide 1) compared to house keeping controls was identical using PCR and microarray techniques (data not shown).

### Within Species Functional Assessment of Osteosarcoma Gene Signature

Using Expression Analysis Systematic Explorer (EASE) analysis [21] we linked each species-specific osteosarcoma gene signature to descriptors of gene function/ontology. The osteosarcoma signature was most significantly associated with extra cellular matrix, structural components, calcium ion binding elements and skeletal development processes (Table 1). Significant functional association with morphogenesis and development (proto-oncogene contribution) was also seen.

**Table 1:** Lowest EASE Scores in Canine Osteosarcoma and Associated Functions.

GO FUNCTION	CATEGORY	LH^2^	EASE SCORE^1^	FISHER EXACT
Cellular Component	ECM	33	5.7E-11	1.09E-12
Molecular Function	ECM structural	16	9.56E-10	8.07E-11
Molecular function	Structural molecule activity	40	0.00000319	0.000013
Molecular function	Ca ion binding	30	0.000132	0.0000565
Cellular component	Extracellular	49	0.000192	0.000105
Biological process	Development	60	0.000227	0.000135
Cellular component	ER	32	0.000285	0.000131
Molecular function	Protein binding	72	0.00403	0.000261
Biological process	Morphogenesis	41	0.000431	0.000228
Biological process	ER to Golgi transport	6	0.00476	0.0000344
Cellular component	Actin cytoskelton	17	0.00354	0.00141

^1 ^Categories with significant Expression Analysis Systematic Explorer (EASE) scores (<0.001) are presented here.

^2 ^LH represents number of genes in gene list assigned to a category.

### Comparison Of Gene Expression In Human And Canine Osteosarcoma

**Figure 2:**
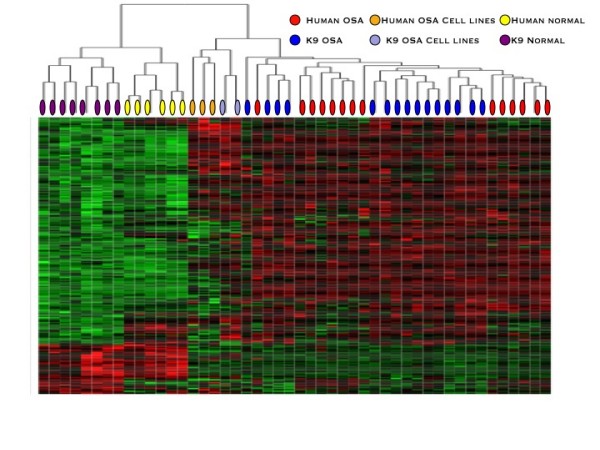
**Cross species analysis of canine and human osteosarcoma are not distinguishable by global gene expression signature**. Comparative genomic analysis was performed by defining the differentially expressed genes between osteosarcoma and normal tissues (adjusted p value < 0.01) and by establishing orthologues between species. After Entrez Gene ID alignment, 265 genes were used to cluster the human and canine osteosarcomas, normal tissues and cell lines. Hierarchical clustering resulted in complete branching of normal and tumor samples, and normal organs could be further defined based on species of origin. Among the 30 primary tumor samples, branching of human and canine osteosarcoma is not divided by species. This suggests that similarities in gene expression signatures in osteosarcoma are due to shared biology across species.

Comparative genomic analysis was performed with orthologues between species osteosarcoma gene expression profiles (i.e., osteosarcoma versus normal tissues (adjusted p value < 0.01). After Entrez Gene ID alignment, 265 genes were used to cluster the human and canine osteosarcomas, normal tissues and cell lines. Hierarchical clustering resulted in complete branching of normal and tumor samples, and normal organs could be further defined based on species of origin. (Figure 2) Among the 30 primary tumor samples there was no distinct branching of human and canine osteosarcoma, suggesting that similarities between cancers in both species was high.

### Canine osteosarcoma as a model of progression

**Figure 3:**
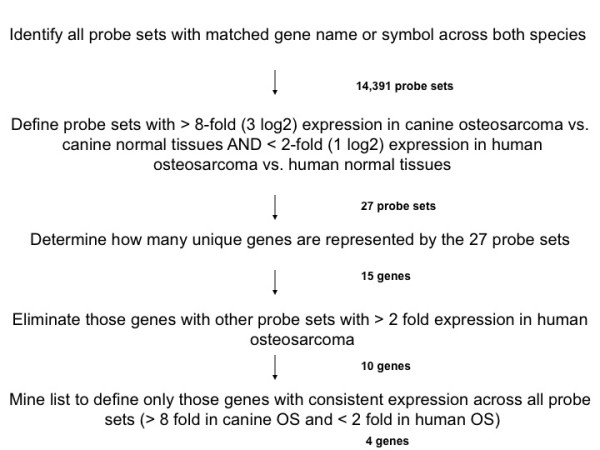
**Algorithm depicting the selection process for dog specific osteosarcoma genes using a fold-expression methodology**. In order to define a list of dog specific osteosarcoma genes that are variably expressed in human osteosarcoma, probe sets with matching gene names or symbols across both species were evaluated (14,391 probe sets). An initial list of dog osteosarcoma defining genes was generated by identifying those probe sets with the highest fold expression differentials between the canine tumors and their normal tissues and present expression in the human tumors and their normal tissues (dog: > 8-fold up-regulation in tumors versus normal; human: <2-fold upregulation in tumors versus normal). This yielded 27 probe sets, representing 15 unique genes. Those genes that also had representative probe sets upregulated in both dog and man (> 8 fold expression) were then excluded, leaving 10 genes. This was further filtered by retaining only those genes with consistent expression across all their Affymetrix probe sets; using these stringent criteria 4 dog-like specific osteosarcoma genes were defined.

**Figure 4:**
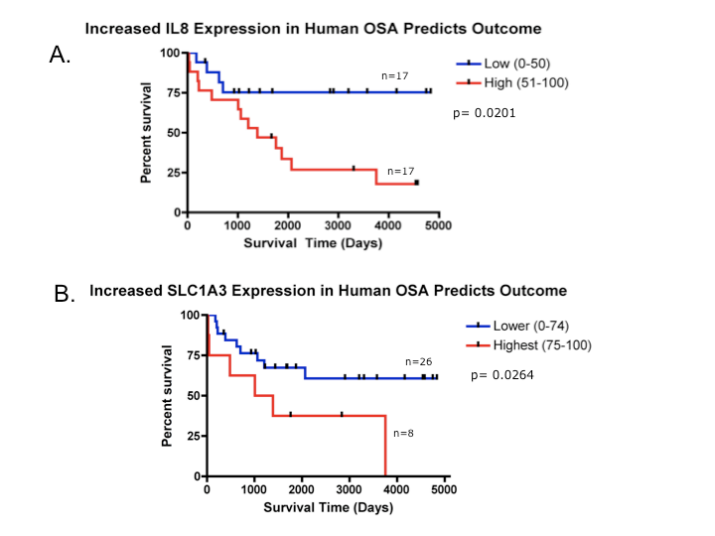
**Canine osteosarcoma can predict genes linked to an aggressive phenotype in human osteosarcoma**. High expression of two of four "dog-like" genes A. IL-8 (p = 0.0201) and B. SLC1A3 (p = 0.0264) were linked to poor outcome in a distinct population of 34 human osteosarcoma patient samples using Kaplan Meier analysis. IL-8's impact on outcome was evaluated according its median expression (Low (0-50) equivalent to < median expression; High (51-100) equivalent to > median expression); whereas SLC1A3 was assessed according to quartile expression (Lower (0-74) equivalent to < highest quartile expression; Highest (75-100) equivalent to highest quartile expression).

The compelling likeness between canine and human osteosarcoma expression profiles prompted us to ask if new insights could be gleaned using our cross-species approach that would be overlooked if only the human data set was examined. Based on the fact that canine osteosarcoma may be associated with a more aggressive clinical course than human osteosarcoma, we hypothesized that genes that were more "dog-like" but nonetheless expressed in human osteosarcoma, would be more likely to be linked to metastatic progression or poorest clinical outcome in human patients. Using a fold expression approach we identified 74 genes with greater expression in the dog vs. human. From this list 15 strongly "dog-like" osteosarcoma defining genes (Table 2) were highly linked to dog osteosarcoma (highest fold expression difference between canine tumors and canine normal tissues; > 8-fold expression in tumors versus dog normals) but were nonetheless expressed in human osteosarcoma (expressed in human tumors compared to human normal tissues; <2-fold expression in tumors vs. normal) (Figure 3). Next, expression consistency in all Affymetrix probe sets, defined as concordant direction of differential expression for all probesets associated with a given gene, yielded four "dog-like" genes of interest: *IL-8*, *SLC1A3*, *TFPI2*, (tissue factor pathway inhibitor 2), and *RBP4 *(retinol binding protein 4, plasma). Each of these "dog-like" genes were examined in a distinct population of 34 human osteosarcoma patient samples (see Additional File 1, Table S1) that had previously undergone gene expression analysis and were linked to clinical outcome. The median survival time for this population of human patients was 10.3 years (see Additional File 1, Table S1)). High expression of two of the four "dog-like" genes (*IL-8 *and *SLC1A3*) linked to poor outcome in human osteosarcoma using Kaplan Meier analysis (*IL-8*, p = 0.0201; *SLC1A3*, p = 0.0264, Figure 4). This finding of two potentially informative genes is noteworthy since the survival signature generated via Cox Regression of the entire dataset suggested it was unlikely that individual genes would be predictive of clinical outcome (data not shown). Fisher exact analysis supported the strength of the association between these "dog-like" genes and survival when compared to 24 previously identified cancer candidate genes (p-value = 0.0189) where no association with survival was found. After multiple test corrections, using random permutation testing, increased *IL-8 *expression was continuously linked with poor survival (p = 0.02). (Figure 4).

**Table 2:** List of twenty-seven probe sets with increased expression in canine osteosarcoma.

Gene Symbol	Gene Name	^1^Canine Tumor: Normal	^2^Human Tumor: Normal
COL1A1	collagen, type I, alpha 1	7.55	-1.00
PTN	pleiotrophin (heparin binding growth factor 8, neurite growth-promoting factor 1)	5.36	-1.01
FN1	fibronectin 1	4.74	0.45
FN1	fibronectin 1	4.74	0.33
DPT	dermatopontin	4.59	0.59
TFPI2	Tissue factor pathway inhibitor 2	4.57	-0.35
TFPI2	tissue factor pathway inhibitor 2	4.57	-0.60
MAP1B	microtubule-associated protein 1B	4.34	-0.14
LAMA4	laminin, alpha 4	3.77	0.49
LAMA4	Laminin, alpha 4	3.77	-0.82
LAMA4	laminin, alpha 4	3.77	-0.58
LAMA4	laminin, alpha 4	3.77	-0.89
SFRP4	secreted frizzled-related protein 4	3.75	0.85
FN1	fibronectin 1	3.59	0.45
FN1	fibronectin 1	3.59	0.33
C1orf21	chromosome 1 open reading frame 21 /// chromosome 1 open reading frame 21	3.58	-1.02
LOXL2	lysyl oxidase-like 2	3.42	-0.56
LOXL2	lysyl oxidase-like 2	3.42	-1.19
FLJ23191	Hypothetical protein FLJ23191	3.36	0.34
RBP4	retinol binding protein 4, plasma	3.28	-1.45
RBP4	Retinol binding protein 4, plasma	3.28	-0.91
IL8	interleukin 8	3.17	0.20
IL8	interleukin 8	3.17	-0.24
SLC1A3	solute carrier family 1 (glial high affinity glutamate transporter), member 3	3.15	0.83
CASK	calcium/calmodulin-dependent serine protein kinase (MAGUK family)	3.07	0.78
FN1	fibronectin 1	3.05	0.45

^1 ^Expression values represent log2 ratios of canine OS samples:canine normal tissues

^2 ^Expression values represent log2 ratios of human OS samples:human normal tissue

To validate the relevance of *IL-8 *and *SLC1A3 *in human osteosarcoma, we evaluated their protein expression in a third and distinct set of human osteosarcoma tissues by tissue microarray (TMA) immunohistochemistry, obtained from primary biopsy, definitive resection and metastases. Results show low to moderate tumor cell expression intensity for IL8 and *SLC1A3 *in most samples (66/67 (98%) and 54/64 (84%) samples have intensity scores <3 respectively) (see Additional File 1, Figure S1). *SLC1A3 *exhibited cytoplasmic staining in tumor and stromal cells; whereas IL8 staining was found in both osteoclasts and osteoblasts.

## Discussion

Our cross-species genomic analysis of osteosarcoma is the first to compare global gene signatures of any spontaneously occurring disease state with the same disease in humans. Two important findings have emerged from this work: (1) there are very strong similarities in gene expression patterns between canine and human osteosarcoma. These similarities further support the inclusion of pet dogs as a translational model in studies of osteosarcoma therapy; and (2) that these similarities, coupled with the more aggressive biology of the canine disease provides a new perspective from which genes and pathways integrally related to the metastatic biology of this disease may be assessed. Indeed, we identified four specific genes that were defined as "dog-like" genes to be expressed in humans. Expression levels of two of these "dog-like" genes (*IL-8 *and *SLC1A3*) were associated with poor outcome in human osteosarcoma patient samples. It is unlikely that these potential progression associated genes would have been considered without the perspective provided by the cross-species approach.

The public release of a high-quality sequence covering 99% of the canine genome (2.5 billion base pairs) has confirmed remarkable similarities between the genomes of dog and man and has provided the opportunity for the cross-species studies conducted here [18,19]. The implementation of such approaches is feasible through commercially available canine oligonucleotide and SNP arrays, first by Affymetrix and now by others. It is now possible for high throughput interrogation of canine tissues and disease states, using platforms and processes previously limited to the mouse and human [4,18,19,22]. Work by several groups has begun to use the canine genome as a means to understand basic biology and genetics of health and disease, including cancer [23-25]. Past comparative genomic studies in cancer have included work in rodents and humans [26,27]. There are many challenges to cross-species comparative genomics that include the dog, including incomplete gene annotation and the lack of methodologies for orthologous gene assessment. We used a comparative genomics methodology, Entrez Gene ID alignment, similar to that described by Sweet-Cordero et al. and Lee et al. to study gene signatures in murine models of cancer, in our canine-human analysis of osteosarcoma progression. Improved cross-species genomic analyses will be possible with further genome annotation and sequence similarity assessments [28].

Past studies that have sought to define targets linked to metastatic progression in osteosarcoma, a highly metastatic pediatric malignancy, have been hampered by the overwhelming bone signature of osteosarcoma, heterogeneous and karyotypic complexity of the disease, relative paucity of tumor samples available before exposure to chemotherapy, and the small number of patient samples available given the rarity of this disease. Baird et al., used expression profiling to define and distinguish osteosarcoma from other similar pediatric cancers, but this signature was highly influenced by its association with bone [29]. Since initial response to neoadjuvant chemotherapy is a recognized prognostic factor in osteosarcoma. Mintz et al. and Ochi et al. used expression analysis by microarray to define classifiers of a poor response to chemotherapy [30,31]. The starting material for these studies were tumor samples obtained following chemotherapy at the time of definitive resection of the tumor [30]. The greater number of dogs who are diagnosed yearly with osteosarcoma, along with the more accelerated progression to metastasis compared to pediatric osteosarcoma, provides a greatly needed resource for the study of this rare pediatric cancer and may be necessary for optimal progress to be made.

Using cluster analysis of human-dog orthologous genes that were differentially expressed between canine cancers and canine normal tissues we were unable to segregate the gene expression signatures of canine from human osteosarcoma. Previous candidate studies of the genetics and biology of osteosarcoma in both species have been limited but supported the similarities between the diseases. We were nonetheless surprised that the expression of orthologous genes could not distinguish the cancers by species. It is important to note that the normal canine and normal human tissue expression signatures were clearly distinguished using this same approach. These data suggest that the osteosarcoma gene expression pattern was dominant over the gene expression patterns of species. Since normal bone was not included as a comparator in normal tissue panel for both, it is reasonable that the dominant expression pattern, common to both species is their association to bone. However, the abundantly expressed biological themes found in canine osteosarcoma are similar to the previously published work describing human osteosarcoma [30-33].

Additionally, it is important to note that the canine normal tissue samples used for this cross-species analysis are true biological replicates and not pooled samples or single samples assayed multiple times. This contrasts the normal human gene expression data derived from the online GNF-GEA database that has batch effects that may introduce bias that differs from expression data obtained from canine osteosarcoma, canine normal, and human osteosarcoma tissues. Limitations exist when harvesting normal human tissue for gene expression analysis due to the fact that most, if not all samples, are attained at various times post mortem. The canine normal tissue samples used in this current study were harvested promptly minimizing sample degradation and bias that may negatively influence gene expression quantification. It is possible that previous genomics studies of human osteosarcoma failed to capture the true biological variability between individuals due to normal sample pooling. Therefore, the ability to collect individual, high-quality canine normal and tumor samples may unmask superior and distinct relationships and improve upon the opportunity to detect biological differences between normal and diseased tissues.

The strength of the similarities between the dog and human gene signatures allowed us to extend the use of the comparative data to identify progression-associated genes. Since dogs have a more aggressive course of disease than humans we hypothesized that "dog-like" genes may define a more aggressive phenotype of human osteosarcoma that could not be previously identified in human genomic evaluations. By identifying genes with the highest expression in a population of canine osteosarcoma and marginal expression in a population of human osteosarcoma patients, two of four "dog-like" genes, IL8 and *SLC1A3 *(*IL-8*, p = 0.0201; *SLC1A3*, p = 0.0264), were defined that were negatively associated with survival in a distinct human osteosarcoma data set. *IL-8 *is a major mediator of the inflammatory response. It functions as both a chemo-attractant for a variety of cell types as well as an angiogenic factor. Both functions have been mechanistically linked to cancer progression in a number of histologies including human melanoma, breast, prostate, pancreatic, head and neck, bladder, ovarian and colorectal carcinomas [34-42]. In a study by Rutkowski et al., elevated serum levels of a variety of cytokines including *IL-8*, *IL-6 *(interleukin-6), *IL-1 *(interleukin-1) and *TNFR1 *(Tissue Necrosis Factor Receptor 1) were linked to tumor extent and poor prognosis in adult patients with bone sarcomas [37]. *IL-8 *up-regulation has also been implicated as a possible pathway for Doxorubicin resistance in a drug resistant human osteosarcoma cell line (143B-DR-DOX), although its impact in paclitaxel resistance in less clear in other in vitro assessments [43,44]. If indeed an indicator of future progression in human osteosarcoma patients, this gene is of particular interest, as it is a druggable target for inhibition because monoclonal *IL-8 *antibodies are already in clinical development [45]. *SLC1A3 *(also known as EAAT1) is a high affinity glutamate transporter that normally regulates neurotransmitter concentrations, although it has also been found outside of the CNS. It has been linked to motility and is highly expressed in aggressive glioma cell lines versus less aggressive variants [46]. Interestingly, it has been recently described by Kalaiti et al. to be present in MG-63, an osteoblastic osteosarcoma cell line, and functionally can be up regulated by glucocorticoids [47,48]. Therefore it may also have implications in bone pathophysiology and as a target for further evaluation in osteosarcoma. The value of the comparative approach (i.e. search for "dog-like" genes) in the studying human osteosarcoma progression associated genes was supported when the predictive value of the dog-genes was compared to candidate non-dog genes previously linked to cancer biology or progression. Furthermore, the demonstration of *IL-8 *and *SLC1A3 *expression at the protein level in human osteosarcoma patient (TMA) samples validated the potential relevance of this comparative approach across platforms. Future studies will include functional analysis of these poor outcome genes within *in vitro and in vivo *models and evaluation of their expression in larger outcome-linked patient datasets. Such data sets are not currently available but are a focus of work by several collaborating groups.

## Conclusions

In summary, the genetic signatures of canine and pediatric osteosarcoma cluster together and are not divided by species. These data, along with the increased incidence of osteosarcoma in dogs provides additional support for the consideration of dogs as a valuable translational model for the study of this cancer. Close evaluation of specific genes more defining of canine osteosarcoma helped identify new genes potentially associated with poor survival in human osteosarcoma patients. The validation of these potential targets in preclinical models and larger human data sets should be prioritized. Collectively the cross-species comparative approach supports the use of the dog as a model for the study of cancer biology, progression and therapy with the long-term goal of improving clinical outcome for human cancer patients.

## Methods

### Samples: Canine

Fifteen tumors were collected from dogs with primary osteosarcoma undergoing definitive surgery at Colorado State University College of Veterinary Medicine and Biomedical Sciences (CSU). All samples were collected prior to chemotherapy. Clinical data was available for canine tumor samples, including patient demographics, anatomic location, further adjuvant therapies employed, and outcome. Normal tissue samples (liver, lymph node, kidney, spleen) were obtained from research colony dogs at Colorado State University. Two normal organs were used for each tissue type. Tissues were sectioned and flash frozen. The Institutional Animal Care and Use Committee reviewed collection protocols.

### Human

Fifteen tumors were collected from children with primary osteosarcoma undergoing initial biopsy of their primary tumor, prior to neoadjuvant chemotherapy. All samples were obtained with informed consent and institutional review board approval. Clinical data was not available for these human tumor samples. Tissues were sectioned and flash frozen. Normal tissue gene expression (liver, lymph node, kidney) was derived from GNF-GEA data previously published [49]. Two normal organs signatures were used for each tissue type.

### Cell lines

Expression analysis of human (U2, HOS and MG63) and canine (MC-KOSA and BW-KOSA) osteosarcoma cell lines included in gene expression studies.

### RNA Extraction: Tissues and Cell lines

Tumor samples were placed in Trizol reagent at 1 ml/100 mg tissue and then homogenized with a Polytron automated homogenizer after crushing with mortar and pestle. All machinery was cleaned before and after each sample with RNase Away (Invitrogen). RNA was extracted via the manufacturers instructions for Trizol extraction. Tumor RNA was then purified via the Qiagen Mini kit using the Qiagen RNA column. RNA was extracted from 5 × 10^6 ^cell lines plated overnight in 100 × 20 mm tissue culture plates using the RNAeasy Mini Protocol (Qiagen) according the manufacturer's directions. RNA quality was assessed via electrophoresis on an Agilent 2100 Bioanalyzer and only high yielding RNA was used for subsequent gene expression analysis.

### Microarrays Canine

All canine tumor and normal tissue samples and cell lines were prepared for cRNA hybridization via the Affymetrix One-cycle Eukaryotic Target Labeling Assay according to manufacturer's instructions http://www.affymetrix.com. 1 μg of total RNA was used for each sample reaction. cRNA was cleaned and fragmented and hybridized to Affymetrix Canine Genome version 1.0 arrays. All samples were hybridized at the National Human Genome Research Institute (NHGRI) array core facility and batched as a group.

### Human

All human tumor samples and cell lines were prepared in the same manner as above. Once cRNA was cleaned and fragmented it was individually hybridized to Affymetrix Human Genome U133A arrays. All samples were prepared and hybridized at the NHGRI array core facility and batched as a group. Human normal tissue Affymetrix .cel files were obtained from the GNF dataset [49].

### Data analysis

Normalization was conducted within each species independently via Robust Multichip Average (RMA) [50]. Identification of differentially expressed genes was performed via limma (Statistical significance (False Discovery Rate <0.05)). Hierarchical clustering of each species' tumor samples, cell lines and normal tissues was performed individually using Euclidian distance and simple linkage [50].

### GEO Accession Numbers

Gene expression data was submitted to the NCBI Gene Expression Omnibus and are available under the following accession number: Super Series GSE16102 (GSE16087: Gene expression profiles of canine osteosarcoma; GSE16088: Gene expression profiles of human osteosarcoma; GSE16091: Gene expression profiles of human osteosarcoma, set2) http://www.ncbi.nlm.nih.gov/geo/query/acc.cgi?acc=GSE16102.

### Ontology

EASE was used for assignment of functional ontology [21].

### Comparative Genomics

Comparative genomic analysis was performed by defining the most statistically significant differentially expressed probe sets between osteosarcoma and normal tissues within each species (p < 0.01). To make the data comparable, expression values for each gene were scaled to have a mean = 0 and SD = 1. Orthologues between these species were defined by the finding the mean of probe sets mapping to each human Entrez gene ID represented. This analysis resulted in 265 Human Entrez Gene IDs then used for hierarchical clustering analysis of all samples.

### Quantitative RT-PCR

We generated first strand cDNA for canine (MC KOSA, BW KOSA) and human (U2, HOS) osteosarcoma cell lines using Promega reagents and carried out quantitative PCR using the iQ(tm)5 Multi-color real time PCR Detection System with iQ(tm) SYBR Green Super Mix (Bio-Rad). Forward and reverse primers were designed for canine and human galectin 3, MFAP5, PRKDC1 genes. These genes were chosen as representative of those up-regulated in both species or up-regulated in the dog. All primers were designed from mRNA sequences for each species individually and oligos generated by Integrated DNA Technologies, Inc. We used GAPDH (house keeping gene) as an endogenous control in each species. Primer sequences are available upon request. All PCR reactions were carried in triplicate with primers at a concentration of 1:10 and a melting point (Tm) of 60°C. We expressed the relative mRNA levels in cell lines as -ΔΔCt, in which ΔCt is the difference in the threshold PCR cycle (Ct) value of mRNA of the gene of interest and the corresponding control (GAPDH) in each reaction.

### Immunohistochemistry

A human osteosarcoma tissue array was used to assess target protein expression [51]. Sections were deparaffinized with xylene and graded alcohol, and then subject to antigen retrieval with Dako Target Retrieval Solution, pH 9 (Dako, Carpinteria CA) for 20 minutes in a vegetable steamer. Blocking was perfomed with 3% H_2_O_2_. Primary antibody was applied for two hours at room temperature (EAAT1, SC-7757 and *IL-8*, SC-73221, Santa Cruz Biotech) and the reaction detected with LSAB+ or Envision Flex + respectively, according to manufacturer's recommendations (Dako). Slides were dehydrated, and coverslipped. Tissue microarrays were scored by manual inspection at 20 × objective magnification (SH). The intensity of antibody immunoreactivity within tumor cells was scored 0,1,2,3 corresponding to negative, weak, moderate and strong staining for both *IL-8 *and *SLC1A3*.

### Defining Dog Like Osteosarcoma Genes

#### Cox Regression

Dog specific osteosarcoma genes were defined by two methods. In the first (Cox regression), the top 250 dog osteosarcoma genes were generated via a t-test comparing differential expression between canine tumor and normal tissues (adjusted p < 0.005). From this group, the top 5% most differentially expressed genes between canine and pediatric osteosarcoma were defined within the scaled data. These genes were ranked by their correlation with survival based on Cox regression p-values.

#### Fold Expression (Figure 3)

Via a second method (fold expression), a list of dog osteosarcoma defining genes was generated by identifying those genes with the highest fold differential expression between the canine tumors and their normal tissues and marginal expression in the human tumors and their normal tissues (dog: > 8-fold up-regulation in tumors versus normal; human: <2-fold up-regulation in tumors versus normal). Expression change was then examined for all probe sets across all genes and only those genes with consistent expression across all probe sets were kept.

#### Evaluation against outcome

Dog specific osteosarcoma genes defined via both methods were evaluated against survival in a distinct set of 34 pre-treatment human osteosarcoma primary tumors (courtesy of TT, treatments and institutions varied) with known outcome (overall survival times), evaluated on HG 133A oligonucleotides arrays (Affymetrix, Santa Clara, CA). The survival signature of this distinct data set was evaluated via Cox Regression survival analysis by defining the top 100 genes via adjusted p-values and querying the data set for their impact on survival. The impact on survival of individual dog-like genes was analyzed in three ways: median centered (high low), quartile analysis and highest expression vs. three lower groups. P values < 0.05 after multiple test correction were considered significant.

#### Fisher Exact Test

The predictive value of the four "dog-like" genes, identified above was compared to 24 candidate genes previously linked to osteosarcoma biology or progression (ex. *Src*: v-src sarcoma (Schmidt-Ruppin A-2) viral oncogene homolog (avian), *PKC*: Paroxysmal kinesigenic choreoathetosis, *CHEK2*: CHK2 checkpoint homolog, personal communication C. Khanna). Each of the 28 genes were analyzed individually for their statistical impact on survival using the methods described above (resulting in a total of 28 × 3 = 84 comparisons). A Fisher exact t test was then used to determine if the use of the dog comparison was more likely to result in genes associated with clinical outcome in a human osteosarcoma vs. a random sampling of candidate genes previously linked to cancer progression. Two tailed p values < 0.05 were considered significant.

## Abbrevations

*IL-8*: interleukin-8; *SLC1A3*: solute carrier family 1 (glial high affinity glutamate transporter), member 3; *PTEN*: phosphatase and tensin homolog; *Rb*: retinoblastoma; *ezrin: *villin-2; *c-met*: mesenchymal-epithelial transition factor; *erbB-2*: v-erb-b2 erythroblastic leukemia viral oncogene homolog 2, neuro/glioblastoma derived oncogene homolog (avian); *p53*: tumor protein 53; *MFAP5*: microfibrillar associated protein 5; *PRKDC*: protein kinase, DNA-activated, catalytic polypeptide; EASE: Expression Analysis Systematic Explorer; *TFPI2*: tissue factor pathway inhibitor 2; *RBP4*: retinol binding protein 4, plasma; *TMA*: tissue microarray; *IL-6*: interleukin-6; *IL-1*: interleukin-1; *TNFR1*: Tissue Necrosis Factor Receptor 1; *CSU*: Colorado State University; *NHGRI*: National Human Genome Research Institute; *RMA*: Robust Multichip Analysis; *Src*: v-src sarcoma (Schmidt-Ruppin A-2) viral oncogene homolog (avian); *PKC*: Paroxysmal kinesigenic choreoathetosis; *CHEK2*: CHK2 checkpoint homolog

## Authors' contributions

MP carried out the molecular genetic studies, participated in the study's design and coordination, analyzed data, performed the statistical analysis, and drafted the manuscript. SD participated in the design of the study, analyzed data, performed the statistical analysis and helped draft the manuscript. SL and SW participated in the study's coordination and data acquisition. LS and PP helped to conceive of the study. SH participated in the study's data analysis. TT helped conceive of the study and participated in the study's coordination. PM and CK conceived of the study, participated in its design and coordination, analyzed data, and helped to draft the manuscript. All authors read and approved the final manuscript.

## Supplementary Material

Additional file 1 **Supplementary Data. Supplementary figure 1. Expression Intensity of IL8 and *SLC1A3 *in Human Osteosarcoma**. Illustrates the protein expression intensity of *IL*-8 and *SLC1A3 *in a human osteosarcoma data set via TMA (primary biopsy, definitive resection and metastatic patients samples). **Supplementary Table 1. Patient Information for Testing Set of Outcome linked Human Osteosarcoma Expression Data. **Describes the patient information for the outcome linked human osteosarcoma expression data set (TT) used to define prognosis for our dog-like genes. Click here for file
