# Supplementary material for: Canine tumor cross-species genomics uncovers targets linked to osteosarcoma progression
Source: BMC Genomics. 2009 Dec 23;10:625. doi: 10.1186/1471-2164-10-625 (PMC2803201; doi:10.1186/1471-2164-10-625)
Supplement: Additional file 1 — Supplementary Data. Supplementary figure 1. Expression Intensity of IL8 and SLC1A3 in Human Osteosarcoma. Illustrates the protein expression intensity of IL-8 and SLC1A3 in a human osteosarcoma data set via TMA (primary biopsy, definitive resection and metastatic patients samples). Supplementary Table 1. Patient Information for Testing Set of Outcome linked Human Osteosarcoma Expression Data. Describes the patient information for the outcome linked human osteosarcoma expression data set (TT) used to define prognosis for our dog-like genes. [file 1471-2164-10-625-S1.DOC]

#### Supplementary Figure 1. Expression Intensity of IL8 and *SLC1A3* in Human Osteosarcoma.

Tissue microarray (TMA) cores were obtained from primary biopsy, definitive resection and metastases. TMA were scored by manual inspection and the intensity of staining within tumor cells was scored as 0,1,2,3 corresponding to negative, weak, moderate and strong staining. Results show variability in expression for **A.** IL8 and **B.** *SLC1A3* in human osteosarcoma for primary biopsy, definitive resection and metastatic patient samples. Also very high expression (intensity score 3) of either gene is rare in all groups (**A. and B.)** This supports our hypothesis that these genes identified in the dog may give insight into human osteosarcoma.

**Supplementary Table 1. Patient Information for Testing Set of Outcome linked Human Osteosarcoma Expression Data*.**

| Histology | Age at dx | Gender | Survival | Patient Status | Specimen Site | Mets at dx |
| --- | --- | --- | --- | --- | --- | --- |
| Osteo | 14 | F | 25 | D | LNS | N |
| Osteo | 12 | M | 43 | D | Parietooccipital area | N |
| Osteo | 19 | M | 176 | D | Lt femur | N |
| Osteo | 13 | M | 205 | D | Rt Femur/tibia | Y |
| Osteo | 7 | F | 225 | D | Rt femur | N |
| Osteo | 49 | F | 352 | A | Lt distal femur | N |
| Osteo | 24 | N/R | 386 | D | Rt proximal humerus | N |
| Osteo | 17 | M | 483 | D | LNS | Y |
| Osteo | 10 | F | 625 | D | Lt proximal femur | N |
| Osteo | 14 | F | 703 | D | Rt distal femur | N |
| Osteo | 19 | M | 927 | A | Lt proximal femur | N |
| Osteo | 13 | F | 1008 | D | Lt ethmoid sinus | N |
| Osteo | 12 | M | 1024 | A | Lt proximal tibia | N |
| Osteo | 19 | F | 1060 | D | Lt distal femur | N |
| Osteo | 10 | F | 1206 | D | Lt humerus | N |
| Osteo | 10 | N/R | 1221 | A | Lt proximal tibia | N |
| Osteo | 7 | M | 1388 | D | Rt proximal humerus | N |
| Osteo | 10 | F | 1436 | A | Rt tibia | N |
| Osteo | 9 | N/R | 1669 | A | Lt distal femur | N |
| Osteo | 15 | M | 1686 | A | Lt proximal tibia | N |
| Osteo | 5 | N/R | 1758 | D | Rt distal femur | N |
| Osteo | 11 | M | 1870 | A | Rt distal femur | N |
| Osteo | 6 | M | 2065 | D | Rt humerus | Y |
| Osteo | 24 | F | 2835 | A | LNS | N |
| Osteo | 15 | M | 2903 | A | Rt humerus | N |
| Osteo | 10 | M | 3200 | A | Lt tibia | N |
| Osteo | 14 | M | 3303 | A | Rt distal femur | N |
| Osteo | 13 | F | 3574 | A | Rt femur | N |
| Osteo | 17 | M | 3753 | D | Left mandible | N |
| Osteo | 19 | M | 4158 | A | Rt arm | N |
| Osteo | 6 | F | 4537 | A | Lt proximal humerus | N |
| Osteo | 9 | F | 4577 | A | Lt distal femur | N |
| Osteo | 7 | M | 4747 | A | Lt humerus | N |
| Osteo | 6 | M | 4836 | A | Rt distal femur | N |

Evaluable patient information includes: histologic confirmation of disease (Osteo=osteosarcoma), age at diagnosis (yr), gender (M: Male, F: Female, N/R: Not recorded), survival time (in days), patient status at last follow up (D dead or A alive), specimen site (primary anatomic tumor location) and status of metastatic disease at diagnosis (Y: Yes or N: No).

*Treatments and institutions varied among patients but median survival time for the entire data set is 3753 days.
